# Elevación de SPINK2 en leucemia mieloide aguda

**DOI:** 10.1515/almed-2023-0011

**Published:** 2023-02-15

**Authors:** Sümbül Gezer, Zeliha Emrence, Tuğrul Elverdi, Muhlis Cem Ar, Burcu Salman Yaylaz, Ferda Paçal, Ayşegül Ünüvar, Melda Sarıman, Ahmet Emre Eşkazan, Serap Karaman, Ayşe Salihoğlu, Zeynep Karakaş, Neslihan Abacı, Sema Sırma-Ekmekci

**Affiliations:** Department of Genetics, Aziz Sancar Institute of Experimental Medicine, Istanbul University, Istanbul, Türkiye; Istanbul University, Institute of Graduate Studies in Health Sciences, Istanbul, Türkiye; Department of Internal Medicine, Cerrahpasa Faculty of Medicine, Division of Hematology, Istanbul University-Cerrahpasa, Istanbul, Türkiye; Istanbul Faculty of Medicine, Division of Pediatric Hematology and Oncology, Istanbul University, Istanbul, Türkiye

**Keywords:** Leucemia mieloide aguda, inhibidores de la serina proteasa, SPINK2, HUSI-II, AML

## Abstract

**Objectivos:**

La leucemia mieloide aguda (AML, por sus siglas en inglés) es una enfermedad muy heterogénea. Aunque se puede clasificar a los pacientes en grupos de riesgo según sus mutaciones genéticas, el pronóstico dentro de cada categoría varía sustancialmente. Es perentorio identificar nuevos marcadores moleculares de la AML. Recientemente, se ha descrito la elevación del inhibidor de la serina peptidasa Kazal tipo 2 (*SPINK2*) en la AML, habiendo sido relacionada con peores resultados clínicos en metaanálisis, así como en un número limitado de pacientes con AML.

**Métodos:**

Analizamos la expresión de *SPINK2* en 62 pacientes (45 adultos y 17 niños) con AML y en 11 líneas celulares mediante PCR cuantitativa (qRT-PCR). Los niveles de la proteína SPINK2 se determinaron en líneas celulares mediante ELISA.

**Resultados:**

Observamos un aumento de expresión del ARNm de *SPINK2* y de los niveles de la proteína en las líneas celulares de AML (HL60 y NB4), frente a otras líneas celulares (K562, Jurkat y NALM6, MCF7, HeLa, HUVEC, hFOB, 293T, U87). Los pacientes con AML mostraron una expresión elevada de ARNm de *SPINK2* frente a los controles (p=0,004) y esta fue significativamente menor en los pacientes t(8;21) positivos, frente a los pacientes negativos (p=0,0006).

**Conclusions:**

Estos resultados sugieren que el gen *SPINK2* tiene un papel relevante en el desarrollo de la AML. Son necesarios más estudios para evaluar la expresión de *SPINK2* en los pacientes con AML con la mutación t(8.21) e investigar su valor pronóstico en varios subgrupos de pacientes con AML.

## Introducción

La leucemia mieloide aguda (AML), una patología maligna progresiva, es un trastorno de las células madre hematopoyéticas caracterizada por un incremento en el número de células mieloides de la médula ósea y el bloqueo de su maduración. Su incidencia aumenta con la edad [1, 2]. La AML es heterogénea en términos genéticos, biológicos y clínicos. Algunas de las alteraciones genéticas observadas en la AML consisten en alteraciones cromosómicas, tales como la pérdida de parte o la totalidad de chr 5 y ch 7, t(15;17)(q22;q12), t(8;21)(q22;q22.1), inv(16)(p13.1q22)/t(16;16)(p13.1;q22), inv(3)(q21.3q26.2)/t(3;3)(q21.3;q26.2), t(6;9)(p23;q34.1)*,* t(9;11)(p21.3;q23.3) *y* t(1;22)(p13.3;q13.3) [3]. Aparte de dichas alteraciones cromosómicas, las mutaciones más comunes se producen en los genes *FLT3, NPM1, DNMT3A, KIT, CEBPA, RUNX1, IDH1/2 TET2 ve MLL, ASLX1, BCOR, BCORL1, TP53, GATA2, DDX41, KMT2A, SRP72 y STAG2* [4, 5].

*SPINK2* (*HUSI-II, ISK2*), un miembro de la familia de inhibidores de la serina proteasa de tipo kazal, actúa como inhibidor de la acrosina-tripsina. La expresión de *SPINK2* está relacionada con la fertilidad [6, 7]. El gen TIG1 codifica una proteína supresora de tumores regulada por retinoides. SPINK2 afectaba a la actividad del activador del plasminógeno tipo uroquinasa (uPA) regulado por TIG1, que regula la invasión, metástasis y transición epitelial-mesenquimal (EMT) [8]. La expresión de *SPINK2* aumenta significativamente en las células ganglionares de la retina tras un daño en el nervio óptico [9]. La mutación en el gen *SPINK2* incrementa la sensibilidad a los estímulos apoptóticos inducidos por la estaurosporina [10]. La ausencia de *SPINK2* induce una vía similar a la microautofagia en las células germinales [11].

*SPINK2* es uno de los genes que se expresan diferencialmente en las células madre hematopoyéticas primitivas extraídas de sangre del cordón umbilical [12]. En un caso clínico se observó que el gen *SPINK2* se encontraba significativamente elevado en los blastos de la médula ósea CD33+ de un paciente con AML, frente a las células de médula ósea CD33+ normales [13]. La base de datos Gene Expression Omnibus (GEO) ofrece evidencia de la elevación de la expresión del gen *SPINK2* en la AML [14]. Los resultados de este estudio revelaron una vinculación entre la expresión elevada de *SPINK2* y malos resultados clínicos [14]. No obstante, son necesarios más estudios para confirmar dichos datos. El objetivo de este estudio es determinar los niveles de expresión de *SPINK2* y evaluar su valor pronóstico en la AML.

## Materiales y métodos

### Muestras de pacientes

En este estudio retrospectivo, se incluyó a 62 pacientes diagnosticados en la Facultad de Medicina de la Universidad de Estambul, el Departamento de Oncología Hematológica Pediátrica, la División de Hematología del Departamento de Medicina Interna de la Facultad de Medicina de Istambul University-Cerrahpasa. Previa obtención de un consentimiento informado, se recogieron muestras de sangre o de médula ósea para su análisis clínico de 17 pacientes pediátricos (menores de 18 años) y 45 adultos (18–80 años) con AML diagnosticada entre 2013 y 2016. El análisis de expresión de *SPINK2* se realizó en muestras sobrantes de ARN de pacientes, obtenidas para el análisis molecular de genes de fusión. El grupo de control estaba formado por tres pacientes pediátricos (menores de 18 años) y cinco adultos (de entre 26 y 67 años) donantes de médula ósea. Además de los controles sanos, también se formó otro grupo de control con nueve individuos sin leucemia a los que se había sometido a una biopsia de médula ósea para el prediagnóstico de la leucemia. Este estudio fue aprobado por el Comité Ético de la Facultad de Medicina de la Universidad de Estambul (número de referencia: 537, 24/05/2017). Este estudio se llevó a cabo de conformidad con las premisas de la Declaración de Helsinki de 1964 y sus posteriores modificaciones. En la Tabla 1 se muestran las características demográficas de los pacientes incluidos en este estudio.

**Tabla 1:** Características clínicas y biológicas de los pacientes con AML.

	AML pediátrico	AML en adultos
Media de edad, años	9,5 (rango: 1,1–80)	50,44 (rango: 18–80)
Sexo, masculino/femenino	29/33	22/23
Media de recuento de leucocitos, célula/µL	8,600 (rango: 2,100–1,80,000)	311,700 (rango: 1,000–1,80,000)
Clasificación FAB, n		
M0	1	1
M1	3	3
M2	7	5
M3	10	4
M4	12	7
M5	3	3
M6	0	0
M7	0	0
ND	26	22

Mutaciones genéticas, n		

t(15;17)-positivo	12	6
t(8;21)-positivo	11	8
Inv(16) o t(16;16)-positivo	6	4
Negativo	33	27

AML, leucemia mieloide aguda; FAB, French American British; M0–M7, grupo FAB del paciente; ND, no disponible.

### Líneas celulares

Las líneas celulares HL60 (leucemia promielocítica aguda), NB4 (leucemia promielocítica humana), K562 (leucemia mieloide crónica), Jurkat (leucemia aguda de células T) y NALM6 (leucemia linfoblástica aguda) se cultivaron en medio RPMI que contenía 10% de FBS, 2 mM de L-glutamina, 100 unidades/mL de penicilina y 100 μg/mL de estreptomicina. MCF7 (célula epitelial obtenida del área metastásica de la glándula mamaria), HeLa (célula epitelial cervical con adenocarcinoma), HUVEC (célula endotelial derivada del cordón umbilical humano), hFOB (osteoblastos humanos), 293T (célula epitelial de riñón embrionario humano), y U87 (línea de células epiteliales de cerebro humano) se cultivaron en medio DMEM, que incluía un 10% de FBS, 2 mM de L-glutamina, 100 unidades/ml de penicilina y 100 μg/mL de estreptomicina. Se cultivaron en una estufa con un 5% de contenido de CO_2_ a 37 °C.

### Determinación de la expresión de ARNm de *SPINK2* en pacientes con AML y líneas celulares mediante qRT- PCR

Las muestras de ARN se extrajeron de las células con el kit RNA Mini de PureLink (Invitrogen, Carlsbad, CA, EE.UU.) siguiendo las instrucciones del fabricante. La RT-PCR se realizó con el kit Transcriptor High Fidelity cDNA Synthesis (Roche Diagnostic, Mannheim, Alemania) siguiendo las instrucciones del fabricante. El ADNc se sintetizó a partir de 500 ng de ARN en un volumen de reacción de 20 µL.

La cuantificación se llevó a cabo mediante un dispositivo de RT-PCR. Como gen control, se empleó el gen constitutivo de la proteína de unión a TATA (TBP). En la Tabla 2 se muestran las secuencias de los cebadores y los tamaños de los productos de la PCR. Para la reacción de PCR de 20 µL se empleó el kit maestro SYBR Green (Roche Diagnostic, Mannheim, Alemania) y 1 µL de muestra de ADNc. La PCR se realizó en las siguientes condiciones: 60 segundos de pre-desnaturalización a 95 °C, 10 segundos a 95 °C, 20 segundos a 68 °C y 10 segundos a 72 °C (45 ciclos). Tras la amplificación, se utilizó el programa para el análisis de la curva de fusión durante 0 segundos a 95 °C, 10 segundos a 65 °C y 0 segundos a 95 °C. Las diferencias de expresión de *SPINK2* entre los pacientes se analizaron mediante el método ∆∆Ct según la muestra de controles sanos [15]. Los niveles de expresión de ARNm se indicaron como unidades arbitrarias (UA).

**Tabla 2:** Secuencias de los cebadoresy tamaños de los productos de la PCR.

Nombre del gen	Cebador	Tamaño del producto de la PCR
*SPINK2*	Cebador hacia adelante: ACCAGGATGTCCCAGACACT	179 bp
	Cebador reverso: GCCAGTGAAGGTGGTCTCTC	
*TBP*	Cebador hacia adelante: ACTTGACCTAAAGACCATTGCAC	120 bp
	Cebador reverso: CTTGAAGTCCAAGAACTTAGCTGG	

### Determinación del nivel de la proteína SPINK2 mediante ELISA

Las células cultivadas se sembraron con two millones de células en 4 mL de medio que contenía 0,2% de FBS adecuado para el tipo de célula. Transcurridas 48 horas, las células se lisaron en 200 µL de solución de extracción (Tris 100 mM, pH 7,4, NaCl 150 mM, EGTA 1 mM, EDTA 1 mM, Triton X-100 al 1%, y desoxicolato de sodio al 0,5%). La concentración total de proteínas en las células se calculó mediante el ensayo de ácido bicinconínico (Pierce BCA Protein Assay kit, Thermo Fisher Scientific, Waltham, MA, EE.UU). La técnica de Sandwich ELISA se aplicó siguiendo el protocolo para el Kit ELISA para inhibidores de la serina proteasa de tipo kazal 2 (SPINK2) (MyBioSource, San Diego, CA, EE.UU). Las muestras se midieron con el lector de placas ELISA A 450 nm. Los niveles intracelulares de proteína SPINK2 se normalizaron a niveles de proteínas totales. Todas las series de muestras, calibradores y controles se realizaron por triplicado. Se calculó el valor medio de las muestras en las tres series.

### Detección de genes de fusión mediante RT-PCR

Las transcripciones de genes de fusión AML1-ETO asociado a t(8;21), y CBFB-MYH11 con inv(16) y PML-RARA asociado a t(15;17) se detectaron mediante reacción en cadena de la polimerasa con transcriptasa inversa (RT-PCR) [16].

### Análisis estadístico

Los datos se analizaron con el programa SPSS Statistics Program Version 20.0 (IBM SPSS Statistics for Windows, Version 20.0. Armonk, NY: IBM Corp.) y GraphPad Prism 7.0. La normalidad de los datos se determinó mediante la prueba Kolmogorov-Smirnov y la prueba Shapiro-Wilk. Para los datos paramétricos se empleó la prueba *t* de Student para muestras independientes y las pruebas de Kruskal-Wallis y la prueba *U* de Mann-Whitney para los datos no paramétricos. La correlación se analizó con las pruebas de Spearman. Se construyeron la curva Característica Operativa del Receptor (ROC) y el área bajo la curva (AUC) para evaluar el valor de la expresión de *SPINK2* a la hora de identificar a los pacientes con AML de los controles. La supervivencia global (SG) se definió como el tiempo transcurrido desde el diagnóstico de la enfermedad hasta el fallecimiento por cualquier causa o con la censura de datos para los pacientes que permanecían vivos en la última fecha conocida de contacto. El análisis Kaplan-Meier y la prueba de log-rank se emplearon para determinar los índices de supervivencia de los pacientes. Un valor p<0.05 se consideró estadísticamente significativo.

## Resultados

### Expresión de *SPINK2* en las líneas celulares

El nivel de ARNm de *SPINK2* en 11 líneas celulares se determinó mediante qRT-PCR (Figura Suplementaria 1). Observamos que la expresión de ARNm fue superior en las líneas celulares de AML (mediana: 10,5 UA, rango: 8,72–12,32 UA) que en las líneas celulares sin AML (mediana: 0,9 UA, rango: 0.41–3.41 UA) mediante la prueba U de Mann-Whitney (p=0,036). La expresión de ARNm de *SPINK2* en las líneas celulares de leucemia (mediana: 3,4 AU, rango: 2,39–12,32 UA) también aumentaron significativamente, en comparación con las líneas celulares sin leucemia (mediana: 0,7 UA, rango: 0,41–1,32 UA) (p=0,006).

Los niveles de proteínas SPINK2 de las líneas celulares, así como las proteínas secretadas en un medio se determinaron mediante el método ELISA. En la Figura Suplementaria 1 se muestran los niveles intracelulares y extracelulares de proteínas SPINK2. También aumentó significativamente la expresión intracelular y extracelular de SPINK2 en las líneas celulares de AML (mediana: 0,074 ng/mg, rango: 0,95–1,04 ng/mg y mediana: 3,85 ng/mg, rango: 0,05–0,30 ng/mg, respectivamente) con respecto a las líneas celulares de AML (mediana: 0,056 ng/mL, rango: 0,05–0,079 ng/mL y mediana: 0,004 ng/mL, rango: 0-0,02 ng/mL, respectivamente) (p=0,036 y p=0,023, respectivamente).

Se encontró una correlación entre los niveles de ARNm de *SPINK2* y los niveles intracelulares y extracelulares de proteínas (p=0,0002, R=0,935 y p=0,046, R=0,610 respectivamente). También se halló una correlación estadísticamente significativa entre los niveles de proteínas intracelulares y secretadas (p=0,002, R=0,817).

### La expresión de *SPINK2* y su relevancia diagnóstica en los pacientes

**Figura 1:**
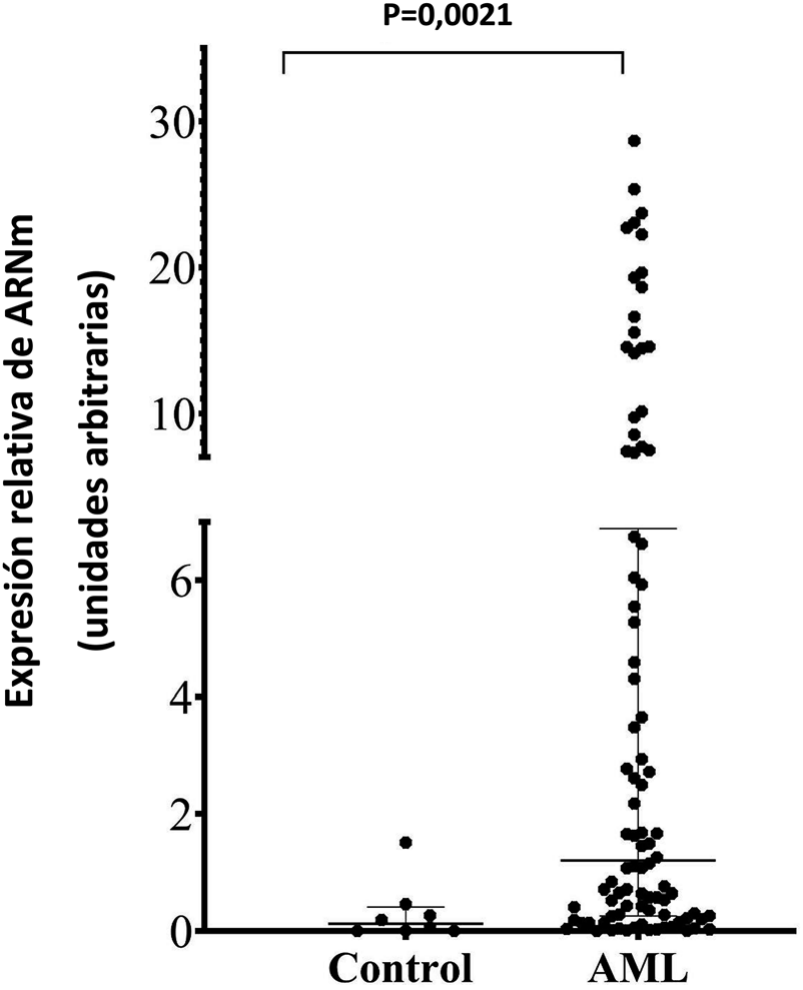
Mediana de nivel de expresión del ARNm de SPINK2 en los controles sanos y pacientes con AML. Los niveles de ARNm de SPINK2 se determinaron a partir de ADNc extraído de 25 ng de ARN total. Los niveles de expresión relativa de ARNm se calcularon empleando el método de CT comparativo. Las líneas horizontales largas sobre los puntos representan la mediana. Las líneas horizontales cortas sobre los puntos representan la mediana de rango intercuartílico. AML, leucemia mieloide aguda.

Los pacientes con AML mostraron niveles de ARNm de *SPINK2* (mediana: 1,62 AU, rango: 0,0032–28,67 AU) significativamente mayores que los controles (mediana: 0,058 AU, rango: 000,017–1,51 AU), lo que indicaba que la expresión de *SPINK2* estaba significativamente aumentada en la AML (p=0,004) (Figura 1). Además de los controles sanos, también se formó otro grupo control con nueve individuos sin leucemia a los que se había sometido a una biopsia de médula ósea para el prediagnóstico de la leucemia. El valor medio de expresión de ARNm de *SPINK2* fue de 0,28 AU (rango: 0,035–1,15 AU). Observamos que SPINK2 estaba significativamente aumentado en la AML, incluso en comparación con las nueve muestras sin leucemia (p=0,022).

**Figura 2:**
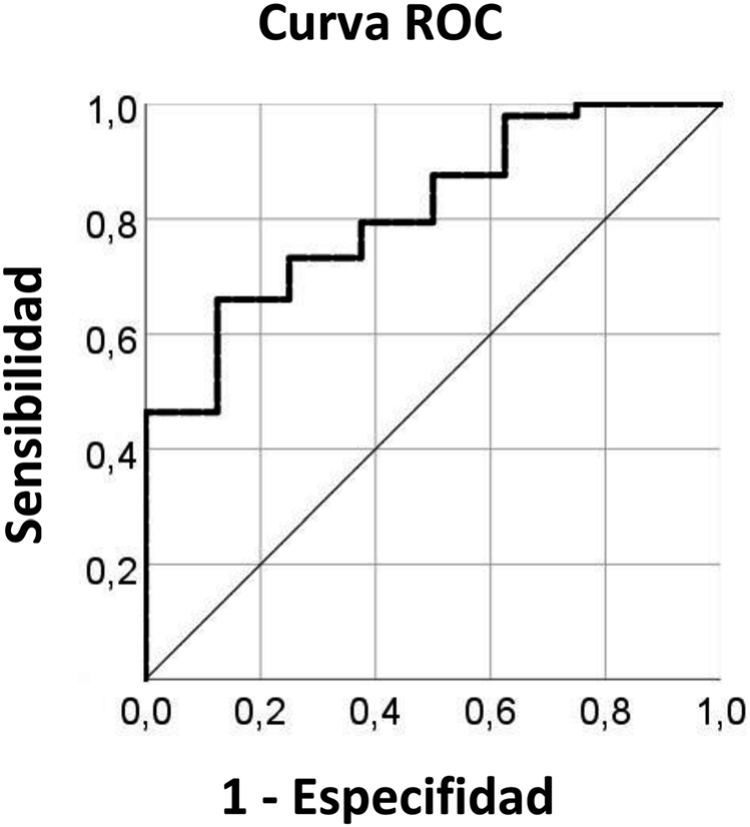
Curva ROC de la expresión de ARNm de SPINK2 en la AML. AML, leucemia mieloide aguda.

Además, se generaron curvas ROC para evaluar el valor diagnóstico de la expresión de *SPINK2* en la AML. El análisis ROC reveló que *SPINK2* es un buen marcador diagnóstico, con un valor AUC de 0,82 [intervalo de confianza al 95% (IC): 0,685–0,946] (p=0,004). El resultado se muestra en la Figura 2.

En la Tabla 3 se compara la expresión de *SPINK2* según las características clínicas y analíticas de los pacientes con AML. No se observó una relación significativa entre la expresión de *SPINK2* y la edad, el sexo, el recuento de hematíes y los grupos French American British (FAB) de los pacientes (Tabla 3).

**Tabla 3:** Comparación de la expresión de *SPINK2* con respecto a las características clínicas y analiticas de los pacientes pediátricos con AML.

	AML pediátrico			AML en adultos		
		n	*SPINK2*		n	*SPINK2*
			p-Valor			p-Valor
Edad, años	10>	10	0,699	60>	31	**0,044**
	10<	7		60<	14	
Leucocitos, célula/µL	10,000>	7	0,527	50,000>	19	0,821
	10,000<	4		50,000<	7	
	ND	6		ND	19	
Sexo	Masculino	7	0,669	Masculino	22	0,946
	Femenino	10		Femenino	23	
FAB	M0	0	0,562	M0	1	0,105
	M1	0		M1	3	
	M2	2		M2	5	
	M3	6		M3	4	
	M4	5		M4	7	
	M5	0		M5	3	
	M6	0		M6	0	
	M7	0		M7	0	
	ND	4		ND	22	
Mutaciones genéticas	t(15;17)	6	0,776	t(15;17)	6	0,317
	t(8;21)	3	0,057	t(8;21)	8	0.00005
	inv(16)	2	0,059	inv(16)	4	0,322
	Negativo	6		Negativo	27	

Los valores en negrita indican que son estadísticamente significativos. AML, leucemia mieloide aguda; FAB, French American British; M0–M7, grupo FAB del paciente; ND, no disponible.

En los pacientes con AML, se analizó la expresión de *SPINK2* con mutaciones t(15;17), (8;21), inv(16) aplicando la prueba U de Mann Whitney (Tabla 3). Se observó una diferencia estadísticamente significativa entre los pacientes t(8;21)-positivos y negativos. Los pacientes con AML t(8;21)-positiva mostraron una expresión significativamente menor de *SPINK2* que los pacientes con AML sin dicha mutación (p=000,006).

**Figura 3:**
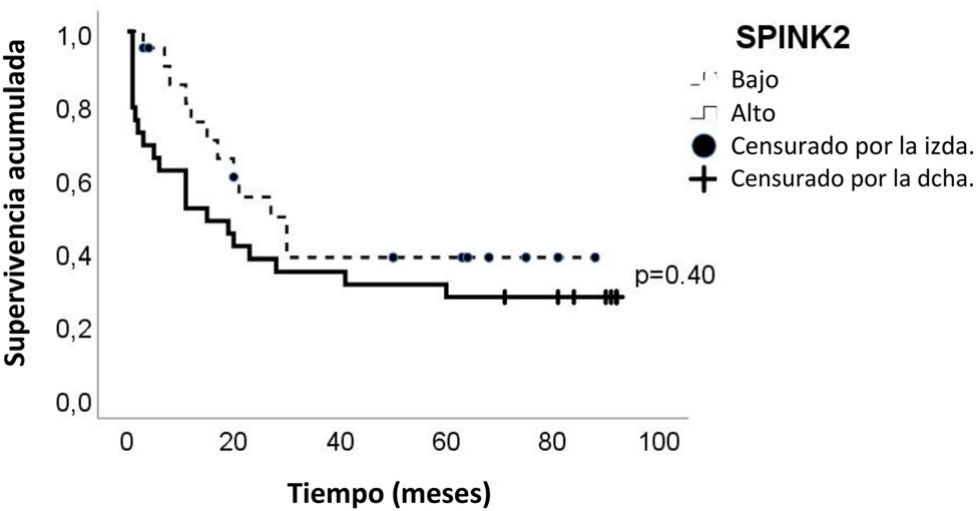
Impacto de la expresión de sobre el índice global de supervivencia de los pacientes con AML. La supervivencia global se evaluó mediante la curva de Kaplan-Meier. El valor umbral se estableció en la mediana de nivel de expresión (1,62 AU) para clasificar a los grupos. AML, leucemia mieloide aguda.

Se clasificó a los pacientes (n=50) en dos grupos (expresión de *SPINK2* ^elevada^ y *SPINK2* ^baja^) según la mediana del nivel de expresión de ARNm de *SPINK2* (1,62 AU) para evaluar el valor pronóstico de la expresión de *SPINK2*. De acuerdo con el análisis de Kaplan-Meier, el índice de supervivencia global a los 7 años fue del 27,9 ± 8,4% para los pacientes con *SPINK2* ^elevado^ y del 40,6 ± 11,6% para los pacientes con expresión de *SPINK2* ^baja^. No hubo diferencias significativas en la supervivencia global entre los pacientes con expresión de *SPINK2* ^elevada^ y *SPINK2* ^baja^ (log-rank p=0.40) (Figura 3). La mediana del tiempo de supervivencia fue de 15 meses (rango: 1–92) para los pacientes con *SPINK2* ^elevada^ y 20,5 (rango: 3–89) meses para aquellos con *SPINK2* ^baja^.

## Discusión

La leucemia mieloide aguda (AML) es una enfermedad heterogénea que causa alteraciones en la hematopoyesis, bloquea la diferenciación y provoca la excesiva proliferación de mieloblastos, así como la interrupción de la producción de células sanguíneas [17]. Aunque la presencia de varias mutaciones se emplea como factor pronóstico para la clasificación de los pacientes, aún hay que afinar la definición de los subgrupos moleculares de pacientes. De este modo, es necesario identificar nuevos marcadores moleculares de la AML.

Existe evidencia de la sobreexpresión de *SPINK2* en las células madre hematopoyéticas [18]. He et al. identificaron *SPINK2* como uno de los genes de expresión diferencial (DEG, por sus siglas en inglés) cuando se comparan las células madre hematopoyéticas CD34+ y CD133+ y las células CD34+ purificadas de sangre de cordón umbilical [12]. El análisis del transcriptoma de los hemoblastos CD33 + BM purificados de un paciente con AML derivado de un síndrome mielodisplásico reveló que la expresión de *SPINK2* era mayor que en las células CD33 + BM de control [13].

En un estudio reciente desarrollado para identificar los principales genes implicados en la AML, se utilizó la bioinformática para analizar los datos de *microarray* del repositorio Gene Expression Omnibus (GEO) Dicho análisis reveló que el gen *SPINK2* era el que mostraba mayores alteraciones de todos los genes que se encontraban sobreexpresados. Dicho hallazgo se validó mediante la realización de qRT-PCR en seis controles y 12 pacientes con AML y, simultáneamente, con datos extraídos de GEPIA (Gene Expression Profilling Interactive Analysis) y Oncomine. Los resultados de este estudio revelaron una vinculación entre la expresión elevada de *SPINK2* y malos resultados clínicos [14]. Barresi y col. observaron que el gen *SPINK2* estaba sobreexpresado en los pacientes NUP98+, empleando datos del repositorio GEO y 358 muestras de AML mediante RNAseq utilizando la base de datos TARGET. Los autores también confirmaron los resultados en pacientes pediátricos con fracaso de inducción primaria mediante qRT-PCR [19].

En nuestro estudio, observamos que el ARNm de *SPINK2* estaba elevado en los pacientes pediátricos, adultos y totales con AML, lo que coincide con los resultados de los estudios de Barresi y col. y Xue y col [14, 19]. El análisis ROC mostró que la expresión de *SPINK2* podía ser utilizada como biomarcador para distinguir a los pacientes con AML de los controles.

La expresión de *SPINK2* se evaluó en los pacientes pediátricos, adultos y totales con AML con mutaciones t(15;17), t(8;21) e inv(16). Los resultados mostraron que la expresión de *SPINK2* se reducía significativamente en los pacientes t(8;21) positivos, frente a los pacientes negativos, tanto en el total, como en los pacientes adultos con AML. Hasta donde sabemos, se trata del primer estudio en mostrar una relación estadísticamente significativa entre la mutación t(8;21) y la expresión de *SPINK2*. Así, es conocida la asociación entre la mutación t(8;21) y un pronóstico favorable [3]. No obstante, Xue y col. relacionaron la expresión de *SPINK2* con un mal pronóstico [14]. Postulamos la hipótesis de que la elevada tasa de supervivencia entre los pacientes con baja expresión de *SPINK2* podría estar relacionada con la positividad de t(8;21). No obstante, son necesarios estudios más amplios para validar la posible asociación entre la expresión de *SPINK2* y la mutación t(8;21).

En este estudio, únicamente se analizó la expresión de ARNm de *SPINK2,* ya que no contábamos con muestras de proteínas de los pacientes. Por otro lado, tampoco pudimos detectar el reflejo de la expresión elevada de ARNm de *SPINK2* en los niveles de proteínas de los pacientes con AML. Analizamos la expresión de *SPINK2* a partir de los niveles de ARNm y de proteínas en 11 líneas celulares, para analizar la posible correlación entre los niveles de ARNm de *SPINK2* y los niveles de proteínas. Los niveles de ARNm de *SPINK2* se determinaron mediante qRT-PCR, mientras que los niveles de proteínas intracelulares y secretadas se determinó en las líneas celulares mediante ELISA. La expresión de ARNm de *SPINK2* en las líneas celulares de AML estaba significativamente aumentada, en comparación con otras líneas celulares. Así mismo, se encontró significativamente aumentada en las líneas celulares de leucemia, frente a las líneas celulares de pacientes sin leucemia. Para comprender las implicaciones de esta molécula en otros tipos de neoplasia hematopoyética, también se debería investigar la expresión de *SPINK2* en la leucemia linfoblástica aguda, el síndrome mielodisplásico y las neoplasias mieloproliferativas.

El nivel de proteínas intracelulares de SPINK2 estaba significativamente aumentado en las líneas celulares de AML, frente a otras líneas celulares. Se observó una correlación estadísticamente significativa entre los niveles de ARNm de *SPINK2* y los niveles de proteínas intracelulares y secretadas. También se halló una correlación estadísticamente significativa entre los niveles de proteínas intracelulares y secretadas. La correlación entre los niveles de ARNm de *SPINK2* y los niveles de proteínas indica que la expresión elevada de ARNm de *SPINK2* aumenta la actividad de las proteínas.

Xue y col. analizaron el efecto del gen *SPINK2* en el pronóstico de los pacientes con AML utilizando datos de GEPIA (106 pacientes) y UALCAN (163 pacientes). Los autores observaron que los pacientes con *SPINK2* ^elevada^ mostraban un tiempo de supervivencia menor que los pacientes con *SPINK2* ^baja^ [14]. En este estudio, no hubo diferencias significativas con *SPINK2* ^elevada^ y *SPINK2* ^baja^ *.* Dado que únicamente se pudo realizar el análisis de supervivencia en un reducido número de pacientes, no se pudieron obtener diferencias estadísticamente significativas.

Este estudio tiene algunas limitaciones. En primer lugar, el análisis de supervivencia se realizó en un número limitado de pacientes, ya que no se disponía de los resultados clínicos de algunos pacientes. En segundo lugar, la expresión de *SPINK2* no se analizó en diferentes subtipos moleculares de AML, ya que no se disponía de los perfiles mutacionales de los pacientes.

Los resultados obtenidos sugieren que la expresión del gen *SPINK2* está elevada en los pacientes con AML, siendo la expresión de *SPINK2* significativamente baja en el subgrupo de pacientes con AML t(8;21) positivo. Es necesario realizar estudios más amplios para determinar el valor pronóstico de la expresión de *SPINK2*. Así mismo, son necesarios más estudios para investigar el posible papel del gen *SPINK2* en el desarrollo de la AML y su uso potencial en terapias dirigidas contra *SPINK2*.

## Supplementary Material

Supplementary Material

## Material Suplementario

La versión en línea de este artículo ofrece material suplementario (https://doi.org/10.1515/almed-2023-0011).
