# Supplementary material for: Elevación de SPINK2 en leucemia mieloide aguda
Source: Adv Lab Med. 2023 Feb 15;4(1):98–104. [Article in Spanish] doi: 10.1515/almed-2023-0011 (PMC10197183; doi:10.1515/almed-2023-0011)
Supplement: Supplementary file 1 — Supplementary Material [file j_almed-2023-0011_suppl.docx]

**Figura suplementaria 1.** Expresión de *SPINK2* en las líneas celulares humanas, incluidas las líneas celulares de AML. (A) Expresión de ARNm de *SPINK2*. Los niveles de ARNm se determinaron empleando ADNc de 25ng de ARN total. Los niveles de expresión de ARNm relativos se calcularon con el método CT. (B) Niveles de proteína SPINK2. Los niveles de proteína SPINK2 se determinaron mediante ELISA. Los niveles intracelulares de proteína SPINK2 se normalizaron por niveles totales de proteína (ng/mg), mientras que los niveles extracelulares de proteína SPINK2 se normalizaron por volumen (ng/mL).

**
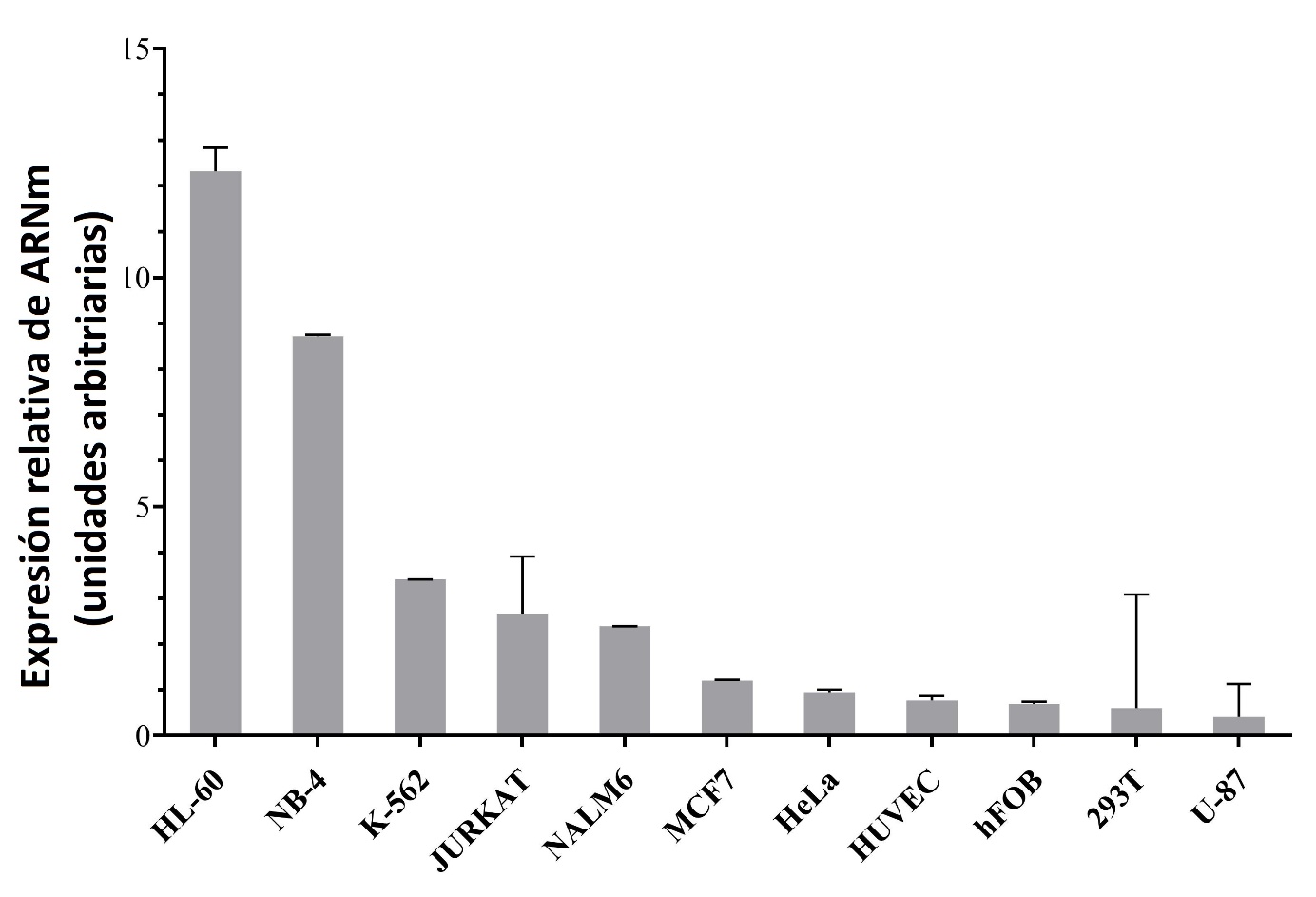

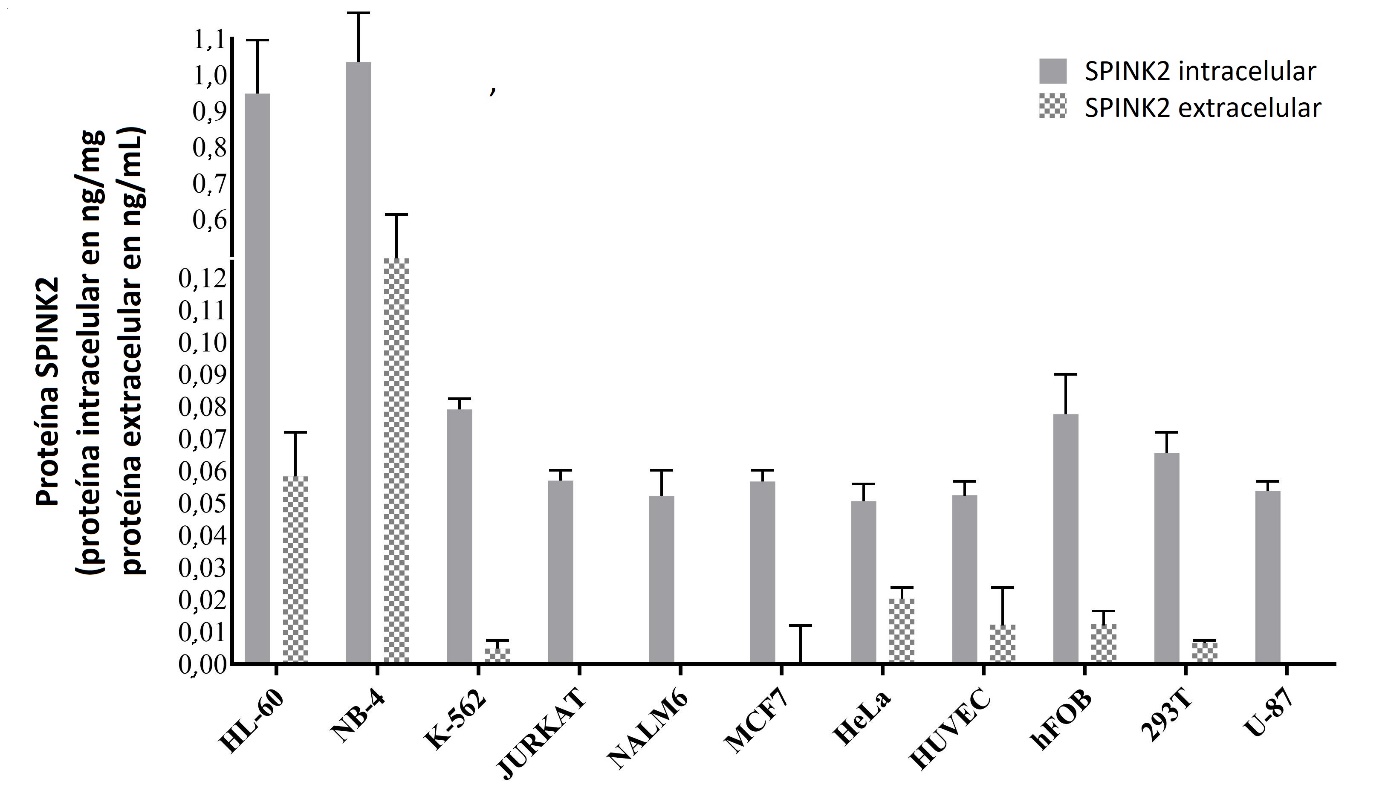
**
